# A qualitative exploration of the emotional wellbeing and support needs of new mothers from Afghanistan living in Melbourne, Australia

**DOI:** 10.1186/s12884-015-0631-z

**Published:** 2015-08-29

**Authors:** Alana Russo, Belinda Lewis, Andrew Joyce, Belinda Crockett, Stanley Luchters

**Affiliations:** Centre for International Health, Burnet Institute, 85 Commercial Road, Melbourne, VIC Australia; Department of Epidemiology and Preventative Medicine, Monash University, 99 Commercial Road, Melbourne, VIC Australia; Refugee Health Program, Monash Health Community, Monash Health, 122 Thomas Street, Dandenong, VIC 3175 Australia; School of Primary Health Care, Monash University, McMahons Road, Frankston, VIC Australia; Centre for Social Impact Swinburne, Swinburne University, John Street, Hawthorn, VIC Australia; School of Health Sciences, Swinburne University, John Street, Hawthorn, VIC Australia; International Centre for Reproductive Health, Department of OBGYN, Ghent University, De Pintelaan 185, Ghent, Belgium

## Abstract

**Background:**

The Afghan community is a priority population for many health and social services within the southeast region of Melbourne, which is home to the largest population of Afghanistan-born people within the state of Victoria. The majority of Afghan women arriving in Australia are of childbearing age, and evidence suggests that they are at increased risk of emotional challenges following birth as a result of the refugee and migration experience. This research aimed to explored the experiences of Afghan women living in Melbourne throughout pregnancy, birth, and early motherhood, and gain insight into the aspects of their experiences that they perceive as positively and negatively impacting their emotional wellbeing.

**Methods:**

This qualitative study collected data through two focus group discussions (conducted in Dari) and 10 in-depth interviews (conducted in Dari or English). Thirty-eight Afghanistan-born women aged 18 years and older, who recently migrated to Australia and have at least one Australian-born child, were purposively selected to participate. A trained bicultural worker assisted in recruitment, data collection and translation. Thematic analysis was performed, and findings were confirmed with a subgroup of participants prior to being included within reporting.

**Results:**

Participants consistently discussed experiencing emotional challenges following birth, identifying symptoms commonly associated with postnatal depression. Women largely attributed this emotional state to separation from family and culture, leading to loneliness, isolation, and disconnection. Participants expressed resistance towards professional support due to cultural stigma associated with mental illness. Partner support was seen to be positive but difficult to negotiate. Religion, strong relationship with child, forming friendships, education, and utilising childcare were identified as positive influences on the emotional wellbeing of women.

**Conclusions:**

This study highlighted social and cultural factors contributing towards the emotional wellbeing of Afghan mothers. Findings confirm the need for innovative community-based models to support the mental health of Afghan women. This is particularly pertinent given the identified resistance towards discussing emotional wellbeing with healthcare professionals. Further research and investment is required in this area.

## Background

The refugee journey frequently includes experiences of torture, forced displacement, separation from friends and family, and extended periods with limited access to healthcare, education, and nutritionally adequate food [1–3]. Settlement within an asylum country presents further challenges related to finance, housing, and difficulties associated with learning a new language and a new culture [2]. Australia settles approximately 13,750 refugees annually under the Australian Humanitarian Program, and it is widely acknowledged that these arrivals experience poorer health than the broader Australian population as a result of complex factors spanning from pre-migration exposures to difficulties encountered within the settlement process [4–6]. In particular, mental health issues are extremely prevalent among this population group, including post-traumatic stress disorder, depression, and anxiety [5, 7, 8].

The majority of women arriving in Australia as refugees are of childbearing age [9]. Therefore, over recent years, there has been an increasing interest in the maternal and child health outcomes and maternity experiences of these women as they have babies, cross-culturally, within Australia. Much of the identified research from Australia has focused on women’s experiences using health services, with the aim of understanding how the system could be improved for women from diverse backgrounds, including refugees [10–13]. Whilst some culturally diverse groups have indicated that existing services devalue their traditional cultural maternity practices [12, 14, 15], other research has suggested that cultural barriers exist for a proportion of women, but ultimately there is an appreciation and acceptance for maternity services within Australia [11, 16, 17]. A common cultural issue is the strong preference to be attended by female health staff and interpreters [11, 13, 18], with additional concerns including negative encounters with staff, inconsistent use of interpreting services, waiting periods, and challenges navigating an unfamiliar health system [10, 17]. While several studies have been framed by a socio-ecological model, and they acknowledge that social and environmental contexts strongly influence individual health, the dominant research focus has been on service access and satisfaction [10, 17]. Consequently, these studies have led to recommendations for system improvement with a view to enhancing accessibility and cultural safety within maternity care. Less emphasis has been placed on the social contexts for women’s mental health during pregnancy, birth and early mothering.

Recent research considering the mental health of new mothers from culturally and linguistically diverse backgrounds suggests that women frequently identify feelings of isolation throughout the perinatal period due to separation from their support networks of other women [17, 19, 20]. Furthermore, literature has suggested that many women of refugee background perceive it to be inappropriate to discuss psychosocial wellbeing with health professionals. They have expressed concerns regarding confidentiality, stigma associated with mental illness, and a perception that this is not an appropriate role of the health professionals they encounter throughout maternity care [17–21].

Since the year 2000, Afghanistan has been a leading country of origin for people arriving and settling in Australia as refugees [22]. Consequently, there has been an increasing interest in the wellbeing of this growing population group, and this has included research on the experience of having a baby within Australia [11, 17]. While aspects of this research have considered social determinants of health, such as social isolation and unemployment, the focus has been set on how this can be addressed through enhanced maternity services. Literature suggests that women with preexisting mental health issues are at increased risk of emotional disturbance following birth, and several studies have indicated that mental health is a significant issue for many Afghan women [23–25]. Therefore, the experiences and emotional wellbeing of Afghan women in Australia throughout pregnancy into the early stages of motherhood are relevant to explore, as pre-migration exposures, compounded by cultural dislocation will profoundly impact their mental health during this period [19].

The majority of research on this topic has explored mental health outcomes and barriers to service uptake, to ultimately enhance service accessibility and acceptability. While increasing accessibility and enhancing acceptability of services is an important strategy, there is a need for an evidence-based approach for community development strategies to promote the emotional wellbeing of these women and other culturally diverse groups. Within existing research, there has been less focus on the broader context of Afghan women’s lives, including how Afghan women explain their own mental health throughout the pregnancy, birth, and early motherhood. Further, there is limited research examining their perceptions of the factors that impact their emotional wellbeing, and potential barriers and opportunities to decrease the risk of mental health problems.

### Theoretical framework

Theory plays an integral role within research; it establishes the lens that is used to critically examine literature, develop research methods, analyse data, and draw conclusions [26]. This research has been framed by feminist and sociocultural paradigms, acknowledging the ways that social and cultural contexts intersect with women’s lives and impact their emotional wellbeing. The combination of feminist and cultural studies frameworks brings and innovative theoretical approach to the study of Afghan mothers’ experiences that has not been widely used in similar studies. However, this approach has been applied in other research focused on culture, parenting and health [27, 28]. Finally, participatory community development values and principles have been considered throughout this project [29]. In particular, this included: recognising the community as a unit of identity; participatory engagement of community members as research collaborators; exploring issues deemed important by the community; and identifying potential opportunities to work collaboratively with the community on these issues.

### Research objective

This research aimed to explore the experiences of Afghan women throughout pregnancy, birth, and into the early stages of motherhood, and gain insight into the aspects of their experience that they perceive as positively and negatively impacting their emotional wellbeing.

## Methods

### Setting

The City of Greater Dandenong and the neighbouring City of Casey are highly diverse municipalities in Melbourne’s southeastern region. The diversity of these catchments has, in part, been nurtured by high rates of refugee and asylum seeker settlement. Whilst refugee settlement data is somewhat limited, it is estimated that 8 % of Australia’s newly arrived refugees settle within this metropolitan region each year, and this figure does not include those who move into the region after living elsewhere in Australia.

The City of Greater Dandenong and the City of Casey are home to the largest settlement of Afghanistan-born people within Victoria, Australia. The majority of Afghans within the region have arrived under Australia’s Humanitarian Program, with additional numbers arriving as part of the family-reunion arrangement. Understanding the experiences and needs of this community has become a priority of local health and social services. The current research was undertaken within these catchments.

### Approach

This qualitative study collected data through two focus group discussions and semi-structured, in-depth interviews. Qualitative methods were particularly well suited to this research given the cross-cultural nature of the study, and the feminist and sociocultural paradigms framing this work [26, 30, 31].

A trained bicultural research assistant partnered with the research team throughout methodology development, recruitment, and data collection. The bicultural research assistant was born in Afghanistan and settled in Australia under the humanitarian program. In addition, the bicultural research assistant is an accredited Dari interpreter. This unique combination of experiences and skills helped to ensure that cultural understanding and appropriateness were inherent within the research questions and process. Furthermore, partnering with this individual assisted in establishing rapport with the community, and lessened language and cultural barriers between researchers and participants.

### Sampling, recruitment, and participants

This research employed a purposive sampling strategy. This method is commonly used within qualitative research as it enables the selection of participants who are particularly well suited to answering the research question [32]. Respondents were recruited to ensure that a broad range of experiences and perceptions were identified; including women who had lived in Australia for varying lengths of time, and with a varying number of children. Further, purposive recruitment ensured the sample had a strong representation of women who had given birth in Afghanistan to enable comparisons to be drawn with the Australian context. To be included in this study, women needed to be over the age of 18 years, originally from Afghanistan and of refugee background. Additionally, participants were required to live in Melbourne, and have arrived in Australia within 7 years from the time of data collection. Participants were required to have a child under the age of 5 years, and have given birth within Australia. Sample size was determined at the point of data saturation. That is, when no new information was yielded from new cases sampled [33].

Women were invited to participate in this research through informal visits to community playgroups that focused on engaging Afghan families in the City of Greater Dandenong and the City of Casey. The bicultural research assistant and the first author attended the playgroups and provided women with an explanation of the project. Researchers presented the option to take part in either an in-depth interview or a focus group discussion. This ensured participants could select a group or private setting to discuss their personal experiences, as felt most appropriate to them. Interested individuals were given a Participant Information and Consent Form (PICF), which was available in English and Dari. This was also offered orally in both languages to compensate for varying literacy levels. All participants were required to provide informed consent, either written or verbally, prior to data collection. Two focus group discussions were conducted within the first phase of the research; 13 women participated within the first focus group, and 15 women participated within the second. Subsequently, 10 in-depth interviews were conducted.

A total of 38 women participated in this research. All participants are of Hazara ethnicity, speaking Hazaragi as their first language which is compatible with Dari. All participants shared the experience of forced displacement, although there was some diversity in their journeys to Australia. Twenty-nine women spent time living in Pakistan before arriving in Australia, seven women came directly from Afghanistan, and two women spent time in Iran before journeying to Australia. Six women arrived in Australia by boat and spent time in mandatory detention. At the time of data collection, participants had been living in Australia for between 1 and 6 years and ranged between 20 and 38 years of age. Only five women identified as being literate in their first language, with three women indicating a degree of literacy in English. Sixteen of the 38 women who participated had given birth and spent time raising children in Afghanistan, and all women had given birth within Australia (either to a first or subsequent child). Participants had between one and four children.

### Data collection

Based on extensive review of the literature, a six-item theme list was developed. This was refined in consultation with the bicultural research assistant to ensure the suitability and cultural acceptability of the content. The theme list included:

- Experiences of being pregnant and giving birth;
- The transition to motherhood;
- Wellbeing and emotions;
- Challenges and supports;
- Relationships and the people around you;
- Comparing and contrasting Afghanistan and Australia.

Prompt questions under each item were developed to be open-ended and non-prescriptive. Interview/focus group outlines were piloted and further refined prior to data collection. The semi-structured nature of the interviews allowed the researcher to ensure that scheduled topics were discussed, while the flexibility of the interview allowed for the emergence of unexpected themes and the discussion of issues of importance to each participant [34].

Both focus group discussions were facilitated in Dari to ensure that all participants felt that they could be involved, were able to follow the entire conversation, and were comfortable within this process. This was maintained by having the bicultural research assistant facilitate the focus groups, with the primary researcher observing and note-taking throughout the sessions. Focus group discussions were conducted in community centres, and were approximately 1 hour in duration. The focus groups were digitally recorded, and then the bicultural research assistant interpreted the recordings, and transcribed them in English.

The in-depth interviews were conducted at locations convenient for the participants. All participants requested interviews to be conducted within their homes. Participants had the option of having the interview conducted in Dari or English, and researchers were allocated according to the preferred language of the participant; six women opted for English and four women opted for Dari. Interviews were up to one and a half hours in duration, as led by the participant, and digitally recorded to allow for subsequent reference and transcription. Interviews conducted in English were transcribed verbatim. Interviews conducted in Dari were interpreted by the bicultural research assistant, and transcribed in English.

The research team has had exclusive access to any identifiable data. Participants’ names have been replaced with pseudonyms to protect their confidentiality. Interview recordings and transcripts have been stored according to ethical guidelines.

### Data analysis

The framework used to guide the thematic data analysis involved five stages; familiarisation, identification of a thematic framework, indexing, charting, and interpretation [35]. The primary researcher led the analysis, with substantial input from the research team at key stages. The analysis developed as an iterative, cyclical, and reflexive process. Preliminary analysis occurred concurrently with data collection; the researchers collected data, transcribed each focus group or interview (replacing all names with pseudonyms for de-identification), and familiarised themselves with emerging themes prior to further data collection [36]. The thematic framework was revised and refined as collection progressed until data saturation was achieved. Focus group and in-depth interview transcripts were manually coded, and crosschecked within the research team. Data were rearranged according to their thematic reference. Key characteristics were drawn together, considering the themes that emerged, linking connections between accounts, and referring findings to the existing literature [35]. The emergent themes were refined and confirmed by the research team.

Consistent with participatory and community development principles, respondent validation was used to ensure that participants were involved throughout the research process [33, 37]. Almost a quarter (*n* = 9) of the women who participated in the research provided feedback on a summary of the findings. All participants were offered the opportunity to contact the research team and be involved in the confirmation process. As no women had taken up this opportunity, the first author and the bicultural research assistant sought feedback from participants via an informal visit to an Afghan community playgroup that was involved in recruitment for the study. Playgroup attendees who had participated in the research were happy to discuss the findings, to confirm that key issues had been thoroughly explored. This process further informed the analysis, and established confirmability.

### Ethics

Given the cross-cultural nature of this research, ethical considerations largely involved ensuring informed consent was obtained. Therefore, the PICF was provided in English and Dari, and was also offered orally in both languages to compensate for varying literacy levels. Participants were provided with multiple opportunities to ask questions prior to providing informed consent. Consent could be provided in written form, or verbally which was digitally recorded. No incentives were offered to any participants within the study.

Ethics approval was granted by Monash Health (previously Southern Health) Human Research Ethics Committee, and Monash University Human Research Ethics Committee.

## Results

Of the many themes that emerged from the data, several have been selected for presentation and detailed analysis based on their relevance to the research question and ability to add to existing knowledge in this area. Themes have been listed under two overarching headings; ‘Experiences within formal maternity care settings’ and ‘Experiences within the context of relationships, home, and community’.

### Experiences within formal maternity care settings

#### Satisfaction with services

The vast majority of participants indicated a high level of acceptance and appreciation for maternity care within Australia, reporting positive experiences with health professionals and the health system more broadly. Participants frequently referenced the maternity care within Australia relative to the absence of formal maternity care within Afghanistan and the poor infant and maternal outcomes experienced there. Overall, participants indicated high levels of engagement and satisfaction with maternity services in Australia.

*“…the health system here is really…very nice*…[in Afghanistan] *most of people just don’t go to doctor…they just fall pregnant and…give birth at home as well…here they do ultrasounds, blood tests, glucose tests, everything was done to take care of me and my child. But there, nothing…”* (Zamira, one child born in Australia, and pregnant with second child. Living in Australia for 5 years)

*“…*[hospital was] *easy and comfortable. I feel free. Everyone very friendly, very happy and helpful..”* (Samila, two children born in Afghanistan, and two children born in Australia. Living in Australia for 4 years)

#### Interaction with health staff

When reflecting on their experiences with maternity services, participants emphasised their interactions with staff to a greater degree than their access to high quality maternity facilities. The majority of women made extremely positive comments about health staff, indicating that they felt respected and included in the decisions regarding their care. Further, several participants identified health professionals as a source of emotional support throughout pregnancy and birth.

*“..*[when I was pregnant]…*the nurse came to me I start crying. I don’t know why and she really listen to me, and she was just so good to me. And after I cried, my heart was like empty. I felt so good and I thought it is just really nice to have someone to understand those kind of situations. I love it.”* (Saera, two children, both born in Australia. Living in Australia for 5 years)

*“..the hard time when I had lots of pain…they help me a lot and hold my hand…I was very happy because they all coming to help me, and it was very good. That’s the thing I can never forget…”* (Batool, one child born in Australia. Living in Australia for 2 years)

Furthermore, a large proportion of participants indicated a willingness to comply with the advice of health professionals, suggesting a high level of acceptance for maternity care practices within Australia.

*“…I cannot ask my mother, because here she hasn’t seen my son, that she could guide me, so it is the midwives that can see and they can guide me. I don’t have experience by myself, but they have knowledge, they have experience, so I was listening to them because I thought that is good for my son…”* (Zamira, mother of one and pregnant with second child)

For some women, this acceptance was double-edged. Women’s confidence in the advice of health professionals was often tempered by beliefs about the importance of advice from their own mothers and other experienced women.

*“…and I was thinking, should I listen to my mum, or to my doctor? I think, my mum is uneducated, doctor is educated, what should I do? First time I was like, sometimes listening to mum, sometimes listening to doctor. The second time all I did* [was] … *listen to my doctor, nothing else. And I did and I find it really good…”* (Shabnam, two children, both born in Australia. Living in Australia for 3 years)

This provides insight into the tensions experienced by Afghan women when seeking support in formal healthcare settings. While they may ultimately ‘accept’ Australian healthcare practices, this often involves a degree of cultural dissonance as women negotiate between competing maternity care practices.

#### Barriers to seeking support in formal settings

A small proportion of women talked openly about their dissatisfaction with the maternity care they received within Australia. Several discussed how their care conflicted with the cultural practices of Afghanistan and identified this as a point of contention in their interactions with staff.

*“Because of the cultural restrictions that I had, I asked to be seen by a female doctor at my antenatal appointments, but the staff got very angry at me and once I had to wait 4 h before I was seen by a female doctor and when I asked them, they said I was being fussy…”* (Feroza, one child born in Australia and pregnant with second child. Living in Australia for 2 years)

“*I felt like I was judged by my doctor…I wanted to do things according to my tradition but I was expected to do things differently..*” (Focus group participant)

Most participants identified a reluctance to discuss their mental health with professionals, indicating that they were not interested in seeking formal psychosocial support, such as counselling, therapy or group treatment. This was primarily attributed to the cultural stigma surrounding mental illness. Further, some women indicated that they did not perceive it to be appropriate for health professionals to assist with emotional support. Expectations that health staff would respond negatively to mental health issues were also a barrier for some participants.

*“..going to some psychiatrist is a very big thing which is not felt good in us…maybe people say that I have gone mad…cause psychiatrist is about mind. If something is wrong in your mind you are mental, you are crazy*… [In] *the* [Afghan] *culture I know, I still believe that it would mean I was going mad…”* (Zamira, mother of one and pregnant with second child)

*“..when the doctor say you need a professional, ah, no, we don’t need…our friend or even our husband look after us…we have to decide what we do…”* (Latifa, one child born in Afghanistan, one child born in Australia. Living in Australia for 6 years)

*“I also had the fear that if I talked to someone that people will come and take my daughter from me because I thought I was going crazy.”* (Feroza, mother of one and pregnant with second child)

### Experiences within the context of relationships, home, and community

#### Family and female kin

Participants consistently talked about the challenges associated with separation from their families, primarily their mothers and sisters, throughout the birth and post birth period. Women discussed their experiences of lacking social support within Australia and the overwhelming majority of women suggested that this contributed towards emotional difficulties in early motherhood.

*“It was hard to be alone here…because I see my sister and my brothers wives in* [Afghanistan] *and there was support from everywhere like family…they get lots of help and the difference only was that I didn’t have my family around me…”* (Saera, mother of two)

*“…you want to cry, why I am alone? My sister was having my mother, why I don’t have anyone?…My mother was going, visiting my sister, and talking, laughing. I was there alone with my son. How can I laugh? How can I smile with him?”* (Zamira, mother of one and pregnant with second child)

Strong family bonds and the importance of these ties between women was a reoccurring theme throughout the narratives. Several participants related the high level of social support within Afghanistan to improved wellbeing outcomes for new mothers within that cultural context.

*“You are close to your mother and your sister like nothing else, not even to your friends. If you give birth…and you can have your mother beside you, you will feel a little bit more comfortable…”* (Latifa, mother of two)

Separation from these supportive relationships was understood as a source of tension, and many women described grief and loss associated with their diluted connection to female kin.

*“If someone is older than you, like your mum or your older sister, if they are around you and they are talking with you, and they are talking about their own experience with you, then you are feeling very better. But I had no one around me, to talk with them…”* (Batool, mother of one)

*“I believe that depression does happen* [to new mothers] *in Afghanistan, but because the family are so strong, people look after each other and women talk to each other. I was left alone to suffer* [in Australia]*.”* (Laila, one child born in Afghanistan, one child born in Australia. Living in Australia for 5 years)

#### Culture, traditions, and community

The importance of traditional practices was a key emergent theme. In particular, women discussed the ways that being separated from these practices in the immediate postpartum period impacted on their experience of having a baby within Australia. Common cultural practices identified as important by women included the clearly defined 10–40 day post-birth rest period, the assistance of female kin and/or a local community birth attendant, ritual celebrations at the end of the rest period, and traditional foods.

*“They* [new mothers in Afghanistan] *were taken care of in very good way. For 40 days, if someone don’t do anything, just take care of the child, then obviously she’s a bit relaxed. Other ladies help them do everything, so she’s relaxed. After 40 days, then she became normal…”* (Zamira, mother of one and pregnant with second child)

*“…people come, dance, eating, everything. They sit over nights to celebrate, especially that happens for the boys…They call the friends, neighbours, everyone they come and enjoy…But here, who will come to sit over night for us? …By that kind of celebrations people do, people come so she* [mother] *feels a bit relaxed, change of mind. That helps a little bit emotionally at that time…”* (Hakima, one child born in Afghanistan and one child born in Australia. Living in Australia for 3 years)

For some women, stories of support and celebration associated with having a baby in Afghanistan were contrasted with descriptions of loneliness and a sense of desertion experienced within Australia.

*“Having a child was such an important time. It was a time when we ate the best food, we get massages every day and were treated with the best of everything. Here we are left to look after ourselves…”* (Focus group participant)

Other traditional Afghan practices mentioned included applying kohl around the infants’ eye, shaving the infants’ head, traditional prayers, and restrictions with hot and cold (foods, ice, and baths/showers). The majority of participants did not find that separation from these cultural practices was difficult for them, with several women indicating that these are easily replicated alone or within the home. However, it was considered important that these practices were recognised by health workers as being legitimate and culturally significant.

Numerous women made reference to the greater sense of community within Afghanistan, compared to Australia. This included increased support from neighbours, less concern about children playing outside, and more assistance with household duties and child minding. This was perceived as supporting the role of mothers within Afghanistan.

*“Motherhood in Afghanistan is easier because your child is not just your child. The whole community, cousins, neighbours and other family look after each other…”* (Focus group participant)

*“I miss my family and I miss the crowds and the neighbourhoods. In Afghanistan, your child is looked after by the entire neighbourhood…”* (Laila, mother of two)

*“…*[in Afghanistan] *the kids can freely play in the streets and mum can sit at home…most of the Afghan ladies* [in Australia] *complain because the kids can’t play outside freely, we have to always sit with them…”* (Saera, mother of two)

*“Raising children by myself is very hard here. If I was back in Afghanistan, my kids would grow up with cousins and I would live with extended family…the work load and responsibilities shared…I am very scared that when my children group up they will have no memories of their culture…”* (Feroza, mother of one and pregnant with second child)

These findings emphasise that cultural practices around birth and early parenting are not only understood as being fundamental to the mental wellbeing of new mothers, but also as an opportunity for sharing and shaping culture with and ‘for’ their young children. For these women, notions of family, community, and a culture of ‘collective caring’ are central to the ways they construct meanings around their own mental health during this stage of their lives.

#### Changing roles of men

Several findings emerged which provided insight into the changing role of Afghan men as husbands and fathers within Australia. Women indicated appreciation for the involvement of men throughout pregnancy and labour, and their increased sense of responsibility as fathers.

*“In Afghanistan the father wasn’t allowed to come close to his wife during labour and birth…in Australia we are very happy that men are told to be there…and they can understand what we go through…”* (Focus group participant)

*“…*[I] *like Australia because the responsibility of raising children is shared* [with husbands] *and fathers are expected to do as much as mothers…”* (Focus group participant)

One of the most prominent emergent themes related to the increased involvement of Afghan men in supporting their wives with emotional challenges following the birth of their child. An overwhelming proportion of participants discussed experiencing emotional difficulties throughout pregnancy and extending into motherhood, with husbands frequently emerging as the predominant source of support for women.

*“I feel sad…I’m crying, I say, ‘I can’t walk, I can’t sleep, I can’t drink, I can’t eat, I, I want to die’…and my husband said ‘if you can’t cook, I cook for you, if you can’t sleep, then sit, or walk’…I’m talk with him…when I say ‘I want to die’, oh he said ‘no…don’t say that, don’t’…”* (Samila, mother of four)

*“I was crying a lot…* [my husband] *was very disturbed because of me. Every time I was crying he said ‘whatsoever you want to do, whatsoever you want from me, I will do, but please just stop crying.’ But I couldn’t make it, I couldn’t stop my crying.”* (Zamira, mother of one and pregnant with second child)

*“He* [husband] *was very nice and taking care of me a lot…he was excited for the baby as well, he was taking care of me because of the baby, because he knew that I am away from my mother and my sister.”* (Batool, mother of one)

Women’s accounts of their husbands’ role during early parenting suggest that new cultural meanings are being generated around fathers’ contribution to emotional wellbeing for new mothers. In addition, women discussed how the emotional challenges they experienced throughout the early stages of motherhood contributed towards stress within their marital relationships.

*“…most of the time I didn’t want to tell anyone that I feel that way* [sad and upset]*. My husband know, he was always with me…I feel like I have no support and because of that I just wanted to fight with my husband, I wanted to empty myself on someone…”* (Saera, mother of two]

*“I just stay at home and think this* [sadness] *will pass. My husband, he is coping with me. Sometimes he gets angry as well…It wasn’t him that wanted the baby, it was me, and now it’s my fault.”* (Hakima, mother of two)

*“I start fighting with my husband. I become angry on him….I used to give him time but I don’t give him now, so I am thinking that have I changed? Whatsoever he say, I start fighting…”* (Zamira, mother of one and pregnant with second child)

These women indicated that they felt disadvantaged by not having adequate support, and this tension was leading them to fight with their partners. Furthermore, despite the shifting and expanding role of men, several women continued to reference the absence of female relationships within their lives.

*“…I only have my husband, but it is not like having an older female in the house.”* (Focus group participant)

For these Afghan women, mental health is understood within the context of their relationships with partners and with other women. These women talked about their own mental health as being strongly linked to the wellbeing of their relationships with their husbands. However, they also described tensions which they believed were due to a lack of support from other women, especially more experienced mothers. These support networks were seen as a buffer which protected their marital relationships and their own emotional wellbeing.

#### Enhancing connection to improve emotional wellbeing

Several participants who identified emotional challenges following birth also identified changes in their lives that improved their emotional wellbeing. Women commonly drew a connection between strengthened social relationships and improved emotional wellbeing after having a baby. Most indicated a general preference to connect with other Afghan women largely due to sharing language. Women who were confident with English were excited to form connections both within and outside of their cultural community.

“*I think that I am like really socialise kind of person, just to have some kind of duty, like to go out…and I experience this when I started my studies, I completely changed my life.*” (Saera, mother of two)

*“I joined a group in my area and within a few months I felt like I was getting better…I realised that a lot of women in the group were having the same emotions like me and that I wasn’t alone.”* (Feroza, mother of one and pregnant with second child)

*“You have to accept the reality. All the time you cannot complain, that why my mother is not here? Why those family friends are not here? You have to accept that this is it, who is here now, you have to relate to them. One of our community friends, she was a bit older than me….My mother said, ‘Now I cannot help you out, whoever is nice to you, that is your kind of mother’…so I accepted this.”* (Hakima, mother of two)

This finding is significant as it emphasises the key role of social and community engagement in supporting the wellbeing of this population group, and reiterates the value of female relationships in promoting emotional health. In addition, several participants discussed how, after a period of sadness, acknowledging their strong relationship with their child ultimately alleviated their sense of isolation and improved their emotional wellbeing.

*“…here I don’t have big groups which I experienced there* [in Afghanistan], so now, for me, it was just my Ali* [son]*. It was not that just I was there for him, he was there for me, so I learned to enjoy that…*” (Hakima, mother of two)

“…*when I saw the face of my child, I think, all the world is mine*.” (Samila, mother of four)

A small proportion of women discussed the key cultural significance of religion within their lives, in particular, how maintaining their connection to religion and performing the associated prayer rituals enabled them to manage their emotional state.

*“I am praying a lot, to keep myself calm, and that’s also helping me. If you are religion, you ask to be calm, not to overreact, and I am praying a lot…whatsoever happen, I will not miss my prayer.”* (Zamira, mother of one and pregnant with second child)

For Afghan mothers, religion and culture are intricately related. This is an important finding as it draws attention to the broader cultural context and the way in which maintaining highly valued aspects of culture can support health within a new country. Finally, participants discussed the importance of designating time for themselves, highlighting the benefit of self-care and relaxation to improving the wellbeing of new mothers.

“*… I didn’t know about this day care…and when I found out, I really loved it, I thought, ‘I wish I knew of this before’. For just a few, 2 h a day or even in a week, just 2 or 3 h…so I can relax…*” (Shabnam, mother of two)

*“Most of the time we really like to have our kids beside us…but sometimes, it is good to have some time without them. That is what I found.”* (Focus group participant)

The findings reported above highlight some of the challenges women experience, whilst offering valuable insights into potential support strategies that women themselves identify as being important.

## Discussion

Within this research, qualitative methods have been used to explore the experiences of Afghan women as they have babies within Australia. This research complements prior studies exploring maternity experiences of Afghan women and families, by extending the focus from maternity care, towards gaining deeper insights into their experiences within relationships, homes and the broader community. These findings confirm the central importance of social and cultural contexts for the emotional wellbeing of new mothers, and identify key opportunities for community development strategies to support the emotional wellbeing of this population.

Prior research has found that Afghan women and families largely view maternity services within Australia as satisfactory and acceptable [11, 17], and the findings of the current study are consistent with this. Furthermore, women within this study placed a high value on the education and advice of maternity care professionals, and this underpinned their willingness to adopt unfamiliar maternity care practices which are common in Australia. While ‘expert opinion’ was met with a degree of resistance by some women, the majority ultimately concluded that maternity care staff provided assistance that enhanced their maternal and child health outcomes and positively impacted on their experience of having a child. This finding contrasts with several earlier studies in which culturally diverse groups have indicated that maternity care within Western culture runs counter to their traditional practices, and adversely impacts their pregnancy, birth and postnatal experience [12, 14, 15]. The cultural acceptability of care was clearly a concern for a small proportion of Afghan women in the current study and, consistent with prior research, this primarily included a preference to be attended by female health staff and interpreters [11, 13, 17, 18, 38]. This confirms the value of continued efforts to enhance cultural safety within healthcare to ensure services are appropriate and acceptable for diverse groups.

While culturally diverse women have been found to highly value Australian maternity care in relation to the physical health of mother and newborn, the findings of the current study highlight a more complex dynamic around maternal mental health. Most women in this research identified emotional challenges that persisted into the months following birth. Participants described experiencing a despondent mood, anxiety, loss of appetite, tearfulness, and feelings of blame and guilt; characteristics which are commonly associated with postnatal depression [39–41]. This is consistent with prior literature which suggests that refugee, asylum seeker and immigrant women are at increased risk of postnatal depression compared to the broader community [19] due to a higher rate of pre-existing mental health conditions, and challenges associated with settling in a new country. The current study confirms that mental health after childbirth is indeed a major issue for Afghan women.

As outlined above, prior research with Afghan women who have given birth in Australia has primarily focused on issues related to access, health service experience, and satisfaction, with the view of enhancing the accessibility and cultural safety of formal maternity care. The majority of women within the current study perceived existing services to be culturally acceptable. However, during the postnatal period and months following birth, women place particular cultural value on a range of community support practices that are considered extremely important for their mental health and emotional wellbeing. These should be central to the process of developing better strategies to help Afghan women cope with the emotional challenges following birth.

Participants identified resistance towards seeking psychosocial support due to cultural stigma associated with mental illness, misunderstandings of how health professionals respond to mental illness, and a broad perception that it is not the role of health professionals to address emotional wellbeing. Similar findings have been identified in prior research [17–21], confirming that barriers exist to addressing mental health issues within a formal healthcare setting. This reiterates the need for innovative, community-based models to support the emotional wellbeing of new mothers from culturally diverse backgrounds, including Afghan women.

This study furthers existing knowledge in the field by offering new insights into the social and cultural contexts of Afghan women’s lives in Australia; the multiple meanings that women construct around mental health within these contexts, and women’s perceptions of their impact on their emotional wellbeing throughout pregnancy, birth and early motherhood. Separation from family, in particular female kin, was a strong theme within this study, and this was perceived as increasing loneliness and isolation during the postnatal period. This has been identified as a contributing factor to postnatal depression among other immigrant groups, who frequently lack social support from family [19].

Mothers in the current study reiterated the fundamental value of other women in providing support for each other throughout the transition into motherhood. Importantly, women also referenced to the shared responsibility and stronger involvement of the community in raising children in Afghanistan, compared to in Australia. This resonates with the culturally inscribed notion that Afghanistan is largely a collectivist society, in which individuals customarily understand themselves and their health within the context of their family and community identity [42]. Dilution of this sense of collective identity, which is fundamental to their culture, is further contributing towards a sense of isolation and disconnection among these women. This finding, which is consistent with prior studies [38], resonates with feminist perspectives on postnatal depression and the mental health and wellbeing of mothers, which considers how social and cultural contexts intersect with women’s experiences of mothering and impact on their wellbeing [40, 43–47]. Afghan mothers face considerable challenges. However, they have their own culturally embedded strategies for addressing their wellbeing needs during pregnancy and early motherhood. Consequently, they are actively engaged in a process of negotiating between Afghan cultural meanings and practices around maternal health and the expert advice and maternity care offered in Australia. The way forward in facilitating improved emotional wellbeing for Afghan mothers is to better understand these strategies, and then do whatever is practicable to further the development and expansion of these in order to enhance their reach and optimise wellbeing outcomes.

This study presents important findings regarding women’s perceptions of the role of men in supporting their mental health. Many culturally diverse groups have indicated that fathers are more involved in pregnancy, birth, and the postnatal period in Australia, compared to their country of origin [16, 17]. This includes attending antenatal appointments, being present throughout the birth, and providing additional assistance with childcare tasks. In prior research, some cultural groups have indicated resistance towards this shift [12]. However, Afghan women within the current study expressed appreciation for the increased involvement of men and identified their potential role as a source of emotional support in motherhood, particularly in the absence of female kin. This study also highlights some of the tensions associated with the cultural transitions within Afghan communities around contemporary fatherhood and their implications for increased stress and conflict within the marital relationship. This finding is pivotal as it emphasises the key importance of developing an understanding of culturally sensitive strategies to assist Afghan men during the transition to fatherhood, and particularly around their role in supporting the emotional wellbeing of their wives.

Women in this research described positive influences on their emotional health; things that made them feel better after a prolonged period of sadness. These are noteworthy as they are united by the overarching theme of enhanced connection (relational, social, and cultural), again highlighting the importance of addressing the broader context of women’s lives to improve emotional health [45]. The most commonly cited reason for feeling better was increased social activity and engagement, including forming new friendships, enrolling in education, and linking into community group programs such as playgroups. Frequently, women transferred family roles to women in these new relationships, referring to the new women in their lives as ‘sisters’ or ‘mothers’, filling the void of absent female kin.

For some women, religion was considered to be a protective factor against social isolation. Maintaining religious rituals and practising valued aspects of culture was seen as a way of promoting better mental health. A prior study identified prayer as a coping strategy for Afghan people [24], while other groups of Afghans have suggested that religion is not important to them [38]. While the Afghan community should not be considered homogeneous, and the needs of community members must be considered at an individual level, this study suggests that encouraging and supporting continued religious practices could be a protective factor for Afghan mothers experiencing emotional challenges.

Additional activity that women considered to be a positive influence on their emotional health included utilising childcare to get some time away from the children, and, conversely, recognising that children can provide a level of company and support for women feeling isolated. This finding is not necessarily contradictory. Rather, both ‘childcare’ and ‘time with children’ were seen as potentially beneficial if they were associated with supportive communities of other women.

Several of the key emergent themes run parallel with experiences of new mothers, and women more broadly. Social support has been widely established as a contributing factor to perinatal emotional wellbeing [48, 49]. In addition, stigma associated with mental health has been found to impact help seeking behaviours among a range of population groups [50–52], including women experiencing postnatal depression [53, 54]. Contextualising the findings of this research within the broader population confirms the need for consistent, population-level strategies to promote the emotional wellbeing of all women. Nonetheless, such issues are compounded for women of refugee background. Displacement results in forced separation from culture and disconnection from traditional social support practices and networks, coupled with language and cultural barriers. These factors have been found to place women of refugee background at increased risk of postnatal depression. Insight into the experiences, practices, beliefs, and understandings of ‘high-risk’ cultural groups can enable a more informed and nuanced approach to working with, and supporting these communities.

The findings of this research are unique as they acknowledge both vulnerability and resilience among Afghan women. They provide insight into their ability to recognise strategies to improve their emotional wellbeing. Furthermore, they confirm the need to support the emotional health of Afghan mothers outside of the formal health setting, and provide pivotal, culturally acceptable leverage points for community development action to address emotional wellbeing among this population group.

### Implications for research and practice

The findings of this research reveal that Afghan women living in Australia face particular emotional challenges surrounding the birth of a child. However, further research into the extent and nature of their mental health needs using both standardised measures and further qualitative inquiry would be beneficial.

There is a need to develop a greater understanding of culturally sensitive strategies to assist Afghan men during the transition to fatherhood, and particularly around their role in supporting the emotional wellbeing of their wives.

Women identified strategies for supporting their mental health, such as increasing social and community connection through forming new friendships, participating within group programs, and partaking in education and employment. Participatory and empowering approaches to community development opportunities that utilise these strategies are highly recommended [46], including developing skills and capacities among Afghan women so that initiatives are driven by the community themselves.

### Strengths and limitations

The qualitative methodology is a primary strength of this research. This approach was particularly well suited given the feminist and cultural diversity principles guiding this work. It enabled the voices of the women to be heard and created a unique opportunity for their personal experiences and perceptions to be explored in greater depth than would have been possible with a quantitative survey. Close collaboration with the bicultural research assistant is an additional strength of this study, as this enhanced the cultural acceptability of the research process and provided unique cultural insights into the project.

There are several limitations to this research. Firstly, the focus groups were relatively large. This did not negatively impact the data, however, there is the risk that not all the participants’ voices were captured in as much detail as would have been possible with smaller groups. Further, due to the cross-cultural nature of this research, a large portion of the data set required interpretation prior to transcription. The bicultural research assistant completing this task holds the qualification of accredited interpreter and underwent additional research training in order to optimise quality and rigour throughout this process. Nonetheless, this contributed an additional layer of interpretation between the raw data and the findings. In addition, the socioeconomic characteristics of participants have not been reported. In future studies, this information would help to contextualise the experiences within individual circumstances. Finally, participants were recruited from community playgroups specifically for Afghan families, meaning that all participants were engaged within a community program. Therefore, it is possible that the voices of the most isolated and vulnerable Afghan mothers were not included within this research.

## Conclusion

This study has highlighted social and cultural factors contributing to compromised emotional wellbeing among Afghan mothers. Findings confirm the need for innovative community-based models to support the maternal mental health of Afghan women. This is particularly pertinent given the identified resistance towards discussing emotional wellbeing with healthcare professionals. By working more closely with women’s own frame of reference, health professionals might make better progress towards developing strategies to support Afghan women’s mental health during pregnancy, early motherhood, and throughout their lives.
